# Do We Need the Environment to Explain Operant Behavior?

**DOI:** 10.3389/fpsyg.2018.00373

**Published:** 2018-03-20

**Authors:** Geir Overskeid

**Affiliations:** Department of Psychology, University of Oslo, Oslo, Norway

**Keywords:** environment, cause, B. F. Skinner, behaviorism, prediction, control

## Abstract

By way of operant conditioning, human behavior is continuously shaped and maintained by its consequences – and understanding this process is important to most fields of psychology and neuroscience. The role of the learning organism’s environment has long been contentious, however. Much relevant research is being done by people identifying with the Skinnerian tradition, who tend to agree that the causes of behavior can be found exclusively in the environment. The meaning of this proposition is not clear, however. Some authors say the environment is outside the body, others claim it is also inside it. Among those who say the environment is outside the body, many are of the opinion that events inside the body and hence (in their view) not in the environment can also cause behavior, though they claim that events inside the body cannot be causes in the same sense as events taking place outside it. This is confusing, and the present paper argues that the “environment” may neither be a useful nor a necessary concept in the analysis of behavior. Moreover, abolishing the concept could clear the way for a reintegration of Skinnerian psychology into the mainstream.

## Introduction

B. F. Skinner (e.g., Skinner, 1981) and those working in the Skinnerian tradition have mapped in great detail how a behavioral repertoire is selected, shaped, and maintained by its consequences. People’s ability to adapt, often unconsciously, to the situations in which they find themselves is based on sensitivity to consequences (Pessiglione et al., 2008; Lieberman, 2012) – and if researchers do not understand how consequences affect behavior, most of what psychology and neuroscience studies will itself be difficult to understand (e.g., Overskeid, 2000). Human behavior is, after all, continuously being affected by operant conditioning, which is, of course, what we call the process by which consequences modify behavior (see Lieberman, 2012).

A wealth of empirical knowledge relevant to operant behavior has long existed, but has not always been integrated into the theories and empirical studies of mainstream psychologists. This, it appears, has to do with the relative isolation of “behavior analysts” (see Overskeid, 1995a), the name often used by those working within the Skinnerian tradition. What, exactly, hinders the integration of this group of researchers into the psychological mainstream, with the potential for dialog and renewed attention to important basic phenomena, like learning and conditioning, that such a development might entail?

## Radical, Yet Increasingly Similar

Behaviorism is more than a century old, though it’s doubtful if anyone now subscribes to the views of Watson (1913), the movement’s founder. B. F. Skinner’s school of thought is another matter (see Overskeid et al., 2012). This American iconoclast once planned to make over “the entire field” of psychology “to suit myself” (Skinner, 1979, p. 38) – and before the cognitive revolution of the 1950s and ‘60s it may have seemed as if he was on his way to doing just that (e.g., de Waal, 2017).

Today, Skinnerian thinking is hardly fashionable. Yet even after his death in 1990, Skinner was still topping a list of the world’s most eminent psychologists (e.g., Haggbloom et al., 2002). A very recent study ranked him second (Green and Martin, 2017). His influence in undeniable, even today, and those working within the Skinnerian paradigm keep producing basic and applied research that is often very relevant to the understanding of operant behavior (e.g., Gomes-Ng et al., 2017; Johnson et al., 2017).

Skinner called himself a “radical” behaviorist – and as opposed to other behaviorisms, the Skinnerian brand fully accepts the existence of private events, like thoughts and feelings. Indeed, Skinner (1974, p. 212) stated: “What is inside the skin, and how do we know about it? The answer is, I believe, the heart of radical behaviorism.” In practice, this has led to cognitive and behaviorist tactics of research becoming increasingly similar when the two schools attack the same problems (see Overskeid, 1995b). However, they do not often work on the same problems. Why is this?

## Separate

There is no doubt that radical behaviorists tend to see themselves as separate from mainstream psychology (Pietras et al., 2013), some even arguing that what they are doing is a separate science, no longer psychology (Vargas, 2017).

What is the basic difference, then, that separates today’s Skinnerian behaviorism from psychology as most psychologists see it – and which still makes it meaningful to speak of a separate school of thought? To Skinner, the assumption was central that the causes of behavior are always to be found in the environment. And Skinner (1984, p. 719) pointed to his “central position” as a reason why psychologists often did not follow his reasoning. “To move from an inner determination of behavior to an environmental determination is a difficult step,” he concluded (Skinner, 1984, p. 719).

Skinner, it seems, hit the nail on its head. The belief in “environmental determination” does indeed appear to be the main theoretical reason why behavior analysis stands apart from mainstream psychology (e.g., Overskeid, 2006), and some have argued that this view of causation is the reason why behavior analysts have been successful in reaching their goals (e.g., Pietras et al., 2013).

So what, then, is the “environment” in behavior analytic theory? What does it determine? And does the belief in environmental determination really hinder the integration of behavior analysis into mainstream psychology?

## Prediction and Control

From its inception, the behaviorist movement has strived to achieve prediction and control of behavior. Watson (1913) was the first to state these goals, and Skinner (e.g., Skinner, 1953) affirmed them. Some behavior analysts prefer “influence” to the word “control” (e.g., Hayes et al., 2013). Skinner, on the other hand, sometimes used a stronger expression, and spoke of “total control” of operant behavior (Skinner, 1986, p. 232). Be the slightly different formulations as they may, the tenet that prediction and control is its purpose “runs through the behavior analytic literature” (Bach and Moran, 2008, p. 18).

The usefulness of an element in a theory, an explanation, or an assumption, must be gaged by the extent to which the element contributes to the attainment of goals – in the case of behavior analysis, prediction and control. At issue, then, is what the concept of the environment can do to help behavior analysts reach their goals.

## Environment and Causes

The environment has been a central concept in behavior analysis. In an oft-quoted passage, Skinner (1957, p. 1) depicts the essence of what behavior analysts analyze: “Men act upon the world, and change it, and are changed in turn by the consequences of their action.” This quotation from Skinner (1957) illustrates that operant behavior is part of a chain of events with no clear beginning or end. Hence those wanting to understand behavior must make certain decisions as to which events should be called “causes,” thus marking them as especially relevant to an analysis having prediction and control as its final goal.

Skinner saw that causes of behavior, that is, events that are not only sine qua non, but also especially relevant to prediction and control, can be found inside as well as outside the body. Indeed, he often emphasized that variations in ease of observation do not create differences in status that are important to the analysis of behavior and its causes. Indeed, “The skin is not that important a boundary. Private and public events have the same kinds of physical dimensions,” said Skinner (1963, p. 953), who was himself a pioneer in the experimental investigation of private events (e.g., Heron and Skinner, 1937).

And in an authoritative exposition of Skinnerian thinking, Delprato and Midgley (1992, p. 1512) concluded: “Private events refer to ‘real’ events, and their ontological status is identical to that of any other aspect of the physical world.” If this interpretation is correct, since private events are identical to other aspects of the world, it should follow that private events can also be causes of behavior, with the same status as any other class of events. Skinner sometimes appears to adopt this view in his theoretical analyses. He says, for instance, that a man may “state his intention,” and explains that “once such a statement has been made, it may well determine action as a sort of self-constructed rule. It is then a true precursor having an obvious effect on subsequent behavior. When it is covert it may be hard to spot; but it is still a form of behavior...” (Skinner, 1969, p. 126).

At other times, Skinner’s appeal to inner causation is by way of illustrations or examples, in which private events more than once are given the status of causes of behavior (for examples, see Zuriff, 1979; Overskeid, 1994). Skinner did make a distinction, however, between causal events that can be observed by more than one individual, and those that are only accessible to the acting person, stating that “private events... may be called causes, but not initiating causes” (Skinner, 1984, p. 719). Behavior analysts appear to agree on this (e.g., Catania, 1988; Pierce and Cheney, 2004).

“Initiating cause” is a term that has been used in many areas of research. If an event in a causal chain can be deemed unusual or conspicuous, and has also appeared relatively close in time to the event to be explained, it is often given the name of an initiating cause (e.g., Sydora et al., 2003; da Silva et al., 2004; Steine et al., 2011). The difference between an ordinary cause and a cause that is not “initiating” has never been fully explained, however (but see Flora and Kestner, 1995, and Overskeid, 2006, for an exchange of views). As regards the present discussion, the important thing is that Skinner clearly, at least from the publication of *Science and Human Behavior* (Skinner, 1953) onwards, saw private events as potential causes of behavior – though not of the initiating type. Private events, according to Skinner, can serve as discriminative stimuli, as well as punishing and reinforcing consequences (for examples, see below, and also Zuriff, 1979; Overskeid, 1994).

## The Role of the Environment

We shall not spend more time discussing external and internal causation *per se*, that has been done elsewhere (e.g., Staddon, 1973; Smith, 1987; Overskeid, 2012). The question is important, however, because it leads directly to the role of the environment in behavior analysis. The environment’s centrality has perhaps been taken for granted, which may be the reason why the concept’s usefulness has hardly been debated – but we shall see that, given the way it has been used by behavior analysts, it may not always be easy to pinpoint the meaning of the word “environment.”

An influential textbook has defined behavior analysis as “the science that studies environmental events that change behavior” (Miller, 2006, p. 5), before going on to explain that “environmental events are any events outside the person.” This may seem entirely reasonable.

Moreover, Skinner appears to agree. In psychology, he explained (Skinner, 1974), several schools of thought have assumed that the environment can exist within a person. The way these schools saw it, “[a] part of the environment entered the body,” said Skinner (1974, p. 73), “was transformed there, perhaps was stored, and eventually emerged as a response.” But behavior analysts, Skinner explained, see this differently: “In an operant analysis, and in the radical behaviorism built upon it, *the environment stays where it is and where it has always been — outside the body*” (Skinner, 1974, p. 73, italics in original).

The Skinnerian point of view is clear, then. Yet it might still lead to difficulties if we consider, for example, the way humans typically perceive their surroundings. Skinner (1953) illustrates this well in his treatment of what he calls the “interpreted” stimulus. A man may think, for instance, that he has found his coat on the coat rack in a restaurant — and given that this is his interpretation of a stimulus, he may start examining the contents of the coat’s pockets, which he would not otherwise have done. Or a person may observe a faint haze at the edge of a forest, and consider whether it is fog or smoke. “[I]n one case we simply pass on; in the other we dash to give the alarm. We may do neither until we have ‘decided which it really is.’ We ‘interpret’ the stimulus before taking specific overt action,” says Skinner (1953, pp. 139–140).

There are, as we saw, many similar examples in Skinner’s writings, but those mentioned here should suffice to show that Miller’s (2006) way of defining “environmental events” and “behavior analysis” runs into difficulties. Though his description of the environment is in agreement with Miller’s, Skinner also describes how responding can be strongly affected by interpretations and other private events — indeed, our interpretation of a stimulus can decide whether we do nothing, or whether we “dash to give the alarm” (Skinner, 1953, p. 139). The interpretation, then, may seem more “initiating” than the external stimulus, described by Skinner (1953, p. 139) as “a faint haze” which in itself does not occasion behavior.

If what Skinner is doing is behavior analysis, these examples alone should show that behavior analysis deals with events that change behavior even if they occur inside a person. Hence Miller (2006) seems to have a problem.

## Outside of Behavior?

A possible solution to Miller’s (2006) predicament is that of Lokke et al. (2011). As opposed to Skinner (1974), they state that seeing the environment solely as existing outside the body is not in keeping with modern behavior analysis, and argue that stimulation from the body as well as consequences within the body are often involved in functional explanations of behavior. It is more precise, say Lokke et al. (2011) to think of the environment as existing outside of behavior, but not necessarily outside the body.

But is this really a solution to our quandary? How easy is it to draw a line between environment and behavior? Can such a line be clearly drawn at all — especially given the fact that behavior analysts typically see behavior as “anything an organism does,” in the words of Catania (1992, p. 364)? Catania goes on to explain that covert behavior is also behavior, and specifies, for instance, that “a shift of attention need not involve eye movements but qualifies as behavior” (Catania, 1992, p. 364).

It is not a controversial assumption that behavior can itself function as discriminative stimuli (e.g., Catania, 1992). Overt behavior can serve this function (e.g., Guerin, 1992), and also private events, as we saw above, as when, for instance, the behavior of interpretation becomes a discriminative stimulus. Skinner (1969) gives many more examples of private rules serving as discriminative stimuli.

It is also well documented that engaging in certain behaviors can function as reinforcement, Premack’s studies (e.g., Premack, 1962) being the most well known demonstrations (see Killeen, 2014, for a more recent discussion of the Premack work). In his 1962 article, Premack concluded (p. 257): “... it was possible not only to reinforce drinking with running, but also to reverse the reinforcement relation in the same subjects...” Zuriff (1979) has an interesting discussion of the several types of covert behaviors that according to Skinner can serve as reinforcement or punishment.

Discriminative and reinforcing stimuli are often seen as being part of the environment, and it is true that they often exist outside the body as well as outside of behavior. It is difficult to claim, however, that this is always the case. Indeed, there is every reason to assume that human behavior is quite frequently under the control of stimuli that are themselves behavior, as when I run because I believe I’m late, and the behavior of running is caused by the behavior of believing. Let’s not quarrel about the exact causal status of believing in this example. Whether one wants to call it an initiating cause or not, it is a cause, and being a behavior, it cannot at the same time be said to exist “outside” of behavior.

Another example: A boy’s doing his homework is reinforced by his parents allowing him to play computer games. The reinforcer, then, is at the same time a behavior, and again something that cannot be said to exist outside of behavior.

If we say that discriminative and reinforcing stimuli are part of the environment, it is not obvious, in other words, that the concept of the “environment” is made more useful by defining it as events taking place outside behavior rather than outside the body.

## Alternatives

It may seem, then, that we are left with two alternatives. The first would be to stick with Skinner’s (1974) and Miller’s (2006) standpoints. However, if the environment exists only outside the body and behavior analysis studies only the effects of environmental events, it is difficult to see how behavior analysis can study covert behaviors like rule following and emotions – even though Skinner (1974) told us above that the what happens inside the skin is the heart of radical behaviorism.

Private rules, for instance, the way Skinner (e.g., Skinner, 1969) saw them, are clearly causes of other behavior (though in his view not “initiating”) — and even a well-known cognitive psychologist has seen Skinner’s theory of rules as “an ingenious analysis” (Sternberg, 1984, p. 605). The second alternative, then, could be to agree with those, including Lokke et al. (2011) who argue that the environment can be inside us – but as we saw above, this, too, may lead to trouble.

A discussion may be needed, then. Do we need to choose between the two alternatives? Should we find a third? Or perhaps one should try to base the behavior analytic search for causes simply on stimuli, the most important being those of the discriminative and reinforcing kind, without necessarily appealing to the environment – a concept that might be superfluous.

It’s not obvious, after all, that prediction and control of behavior is always made easier by including the concept of the environment in any analysis. The discussion above may indicate, instead, that using the word can complicate things. “The point is,” said Staddon (1993, p. 446), “that the environment-based versus organism-based distinction is often impossible to make in practice.”

As opposed to the environment, it is uncontroversial among Skinnerians that *stimuli* can occur inside the body. The stimuli that give rise to seeing are good illustration, as in “Seeing does not require something seen,” Skinner’s famous dictum – after which he continued: “We acquire the behavior of seeing under stimulation from actual objects, but it may occur in the absence of these objects under the control of other variables” (Skinner, 1963, p. 955). “Other variables” are not necessarily outside the body. Indeed, if we close our eyes, and still see an object, our seeing must necessarily occur in the absence of actual objects, and must therefore be occasioned by private stimulation (see Skinner, 1963).

## What is a Private Event?

In behavior analytic terminology, a stimulus or a response is private or covert when it is accessible only to the person whose behavior it affects (if a stimulus) or whose behavior it is (if a response). For stimuli or responses to escape the fate of being called covert, many types of observation appear acceptable, however. A machine can register a rat’s lever pressing, and even if no one watched the rat in its experimental chamber, we regard the machine’s registration as evidence that the behavior has taken place, and do not call it a private event. A fish may swim around in a pond inside a cave that cannot be accessed by humans. We can get a camera into the pond, however, and even though we need the aid of machinery to observe the swimming fish, we do not call its swimming a covert response.

There are now more ways than ever in which machinery can blur the line between public and private, and Skinner pointed out (Skinner, 1989, p. 18) that “[t]here are two unavoidable gaps in any behavioral account: one between the stimulating action of the environment and the response of the organism and one between consequences and the resulting change in behavior. Only brain science can fill those gaps.” Since computer programs using data from brain imaging can now reliably decode things people imagine, intend, and remember (see Smith, 2013), it is getting more difficult, in many instances, to see the difference between public and private events.

As technology continues to advance, it should become increasingly easy to study more directly the private events that radical behaviorists already see not only as real, but even as important aspects of human behavior (e.g., Skinner, 1974). Brain science has come, in other words, some way toward filling the gaps that Skinner (1989) described. Furthermore, improved access to neural processes may weaken the distinction between public and private events, which could make it less meaningful to differentiate between events taking place in or outside the environment. Indeed, “the skin is not that important a boundary,” we saw Skinner pointing out as early as 1963 (p. 953).

## Conclusion

It does not seem obvious that ascribing all causes of behavior to the environment can always help behavior analysts get closer to their goals of prediction and control. Indeed, it is not always obvious what the “environment” refers to in behavior analytic terminology, and whether applying the term can make explanations and hypotheses any clearer. It may be the case, however, that including the “environment” in behavior analytic hypotheses or explanations can sometimes hinder prediction and control. There are two main reasons for this.

First, scientists continue to prefer the simplest explanation that is consistent with existing data (e.g., Gauch, 2003) – whether it be based on simple hypotheses’ greater amenability to testing (see Baker, 2010), or on an assumption that simpler hypotheses have, other things equal, a greater probability of being true (Jefferys and Berger, 1992; Swinburne, 1997). We have seen that if the concept of stimulus is used in an analysis of behavior, introducing the term “environment” is sometimes – perhaps always – superfluous, and therefore contrary to the scientific ideal of simplicity.

Second, if the environment is taken to be the abode of the only stimuli that can initiate responding, this could make researchers look for causes only in those places they regard as parts of the environment – thus running the risk of ending up by manipulating stimuli that do not change behavior in the most efficient manner. It is worth remembering that Skinner (1953, 1969) described how a person’s interpretations, intentions, and other rules can affect his or her behavior in important ways – even if, by some definitions, such private events are not initiating causes.

When private events are important determinants of behavior, it can sometimes be a mistake not to focus primarily on changing those events if the goal is to change the way a person acts. After all, my interpretation of a stimulus can decide if I raise the alarm or do nothing, and following a rule can even make operant behavior insensitive to consequences (e.g., Hayes et al., 1986).

The concept of stimuli – discriminative, reinforcing, or otherwise, is of course as important as always. But causes are everywhere, and their importance does not always depend on their visibility or where they are to be found. It is clearly possible to speak of causes simply in terms of stimuli, and it is not clear that anything would be lost if one stopped referring to the “environment.”

Mainstream psychologists believe that thoughts and feelings are central to the phenomena they study – and so do Skinnerian radical behaviorists. Mainstream psychologists also formulate theories purporting to explain phenomena that cannot be observed directly – and radical behaviorists, too, have done so for a long time (e.g., Skinner, 1969). Still, an important difference is the radical behaviorist belief that “initiating” causes exist only in the environment. Mainstream psychologists do not share this assumption. Might it be possible, then, that if behaviorists were to accept a line of argument like that advanced in the present article, a reintegration into psychology proper could take place? The present author would be tempted to say yes.

The present author might be wrong, however. For instance, there are certain practices and certain areas of research that are quite specific to behavior analysis, even if they do not necessarily depend on theoretical assumptions that are specific to that field. Incentives may exist that preserve such traditions, even if they may not be the most effective way of acquiring knowledge (see Vyse, 2013). This may indicate that a change in theoretical outlook, if it were to happen, would not necessarily lead to a change in practice.

Moreover, it is sometimes said that new ideas are not accepted on account of facts and arguments, but because those who hold the old ideas die out. If there is truth in this, it may be due to social mechanisms such as the shared world view that is typical of many groups (see Peñaloza and Venkatesh, 2006), and cognitive mechanisms like confirmation bias (e.g., Doll et al., 2011) – things that aren’t easily changed. Yet facts are stubborn things – more stubborn, it seems, than human minds. That’s why paradigms do change, after all, and also why debate in science is worthwhile.

## Author Contributions

The author confirmed being the sole contributor of this work and approved it for publication.

## Conflict of Interest Statement

The author declares that the research was conducted in the absence of any commercial or financial relationships that could be construed as a potential conflict of interest.
